# Rationalizing the GMO Debate: The Ordonomic Approach to Addressing Agricultural Myths

**DOI:** 10.3390/ijerph13050476

**Published:** 2016-05-09

**Authors:** Stefan Hielscher, Ingo Pies, Vladislav Valentinov, Lioudmila Chatalova

**Affiliations:** 1 Chair of Economic Ethics, Martin Luther University in Halle-Wittenberg, Grosse Steinstraße 73, Halle 06108, Germany; ingo.pies@ wiwi.uni-halle.de; 2 Leibniz Institute of Agricultural Development in Transition Economies, Theodor-Lieser-Str. 2, Halle 06120, Germany; valentinov@iamo.de (V.V.); chatalova@iamo.de (L.C.)

**Keywords:** agricultural myths, discourse, ethics, GMO, morality, ordonomics

## Abstract

The public discourse on the acceptability of genetically modified organisms (GMOs) is not only controversial, but also infused with highly emotional and moralizing rhetoric. Although the assessment of risks and benefits of GMOs must be a scientific exercise, many debates on this issue seem to remain impervious to scientific evidence. In many cases, the moral psychology attributes of the general public create incentives for both GMO opponents and proponents to pursue misleading public campaigns, which impede the comprehensive assessment of the full spectrum of the risks and benefits of GMOs. The ordonomic approach to economic ethics introduced in this research note is helpful for disentangling the socio-economic and moral components of the GMO debate by re- and deconstructing moral claims.

## 1. Introduction

Public controversy is the new hallmark of the global food and fiber system. Among the hotly debated issues, the acceptability of genetically modified organisms (GMOs) stands out in terms of the heatedness of debates and the concomitant public anxiety. Especially in Europe, the public perception of GMOs appears to be infused with highly emotional and moralizing rhetoric that associates any man-made genome alterations with “Frankenfood” [1] or “ultimate reinforcement of highly industrialized agriculture” [2] (p. 57). The relevant public debates tend to be centered on the potential environmental, social and economic risks of GMOs while turning a blind eye to the significant advances in bioengineering that allow for less invasive genome editing, such as CRISPR and other types of mutagenesis [3,4]. The high publicity of these debates is not at all conducive to their openness toward the potential and already proven benefits of GMOs [5] and, thus, to their ability to account for the full spectrum of dangers and merits of transgenic technologies. 

Until now, the available long-term studies could not find any “validated evidence that genetically modified (GM) crops have greater adverse impact on health and the environment than any other technology used in plant breeding” [6] (p. 2) or that GM foods entail more or different risks for health than conventional ones [7]. The reasons for the public opposition to GMOs, thus, seem to be disconnected from the knowledge base generated by natural sciences. In this line, De Cheveigné and coauthors [8] point out the hesitant political attitude to GMOs that has added confusion to the debate and fueled mistrust toward scientific arguments and regulatory processes. A recent illustration of political hesitation is the decision of seventeen EU countries in 2015 to opt out of the possibility to commercially produce genetically modified food and fiber, without imposing import ban on GM products despite the otherwise acknowledged safety and risk prevention arguments. Furthermore, according to Bonny [2], agricultural overproduction in many European countries renders the potential GMO-induced yield increases small or non-existent while their potential risks are perceived as substantial and irreversible. 

An increasing number of studies in the fields of economics, sociology, and moral psychology [7,9,10] testify to the tendency of public attitudes toward GMOs to be framed not by scientific evidence but rather by individual values and moral traditions. In fact, the GMO debates are not only highly emotional but also deeply saturated with moral content [11]. A similar prominence of moral ideals seems to be characteristic of other agrifood issues, such as food waste, speculation with agricultural commodities, or small-scale farming. Each of these issues identifies social groups on which harm is supposedly inflicted. Possible harmful consequences and possible underlying intentions constitute the moral categories that make public discourse intensely emotional. With some social groups seen as harmed and disaffected, other groups stand up to public scorn and condemnation [12]. 

It is very likely that the prominence of moral ideals gives rise to rigid mental models that are comparable to myths in terms of their resistance to revision. One prominent mental model is what Paul Collier [13] calls “the middle- and upper-class love affair with peasant agriculture”. This “love affair” is widespread despite the fact that the highly productive agriculture in the Western hemisphere is heavily industrialized, and, indeed, it is productive to the extent that it is industrialized. Political instruments aimed at protecting and maintaining small-scale peasant farming carry wide emotional appeal, yet they can present a dysfunctional hindrance on both structural change and productivity in many segments of global agriculture. Essentially similar concerns can be raised with regard to mental models related to GMOs or to speculation with agricultural commodities [14,15]. These models, while seeking to reconcile the reality of industrialized agriculture with sustainability ideals, are apparently impervious to the evidence on how genetic engineering boosts agricultural productivity [16] and how speculation allows small producers to hedge against price risks [15]. In view of their impressive resistance to parts of empirical evidence, it does not seem far-fetched to characterize these mental modes as modern myths which are defined by the Merriam Webster dictionary as ideas or stories that are believed by many people but are not true. Despite their controversial nature, modern agricultural myths exert real effects not only on public discourse but also on the socio-economic well-being of food consumers and food producers alike.

The present research note calls readers’ attention to the “ordonomic approach”, which is a recent strand of economic ethics critically examining the interdependencies between institutions and ideas, or in Niklas Luhmann’s terminology, between social structure and semantics, the former of which means rule arrangements and their incentive patterns, while the latter includes mindsets, worldviews, mental models and other discourse-framing thought categories [17] (pp. 378–388). The key idea of the ordonomic approach is that discrepancies between social structure and semantics can result in misconceiving situations and thus in bringing about social dilemmas marked by collective self-damage. In many cases, the public frame of perception has an extremely narrow focus that overexposes conflicting interests, while it at the same time underexposes the common interests inherent in the situation. Therefore, empirically re-constructing and analytically de-constructing dysfunctional discourse patterns can lead to a shift in perspective that may ultimately prove useful for finding (new) rule arrangements that help to solve the conflict. Such re-framing efforts reflect the normative orientation of the ordonomic approach toward the Rawlsian vision of society as an enterprise for mutual advantage [18]. While following this admittedly weak normative orientation, the ordonomic approach never positions itself within conflictual value discourses but rather seeks to transcend them by realizing mutually beneficial win-win outcomes. The rest of this research note will illustrate the ability of the ordonomic approach to analyze and straighten out the distorted discourses, both in general and in relation to the problem of widespread opposition to GMOs. 

## 2. The Ordonomic Approach 

### 2.1. The Problem of Discourse Distortions 

The constitutive role of discourses for human communication, culture, and civilization is emphasized by modern discourse ethics and poststructuralist philosophy [19]. Both of these literatures admit, however, that real-world discourses may exhibit serious imperfections or distortions that forestall the achievement of an informed rational consensus. Pincione und Tesón [20] go so far as to refer to “discourse failures” that may result “from the combination of the incentive of politicians and lobbyists to spread inaccurate views, the high cost for members of the public to check the credentials of easily available views and the possibility for politicians to access the redistributive apparatus of the modern state”. The authors develop a compelling account of why political actors, including corporations and nongovernmental organizations (NGOs), may systematically take opportunistic advantage of citizens’ rational ignorance and thus gain private benefits at the expense of the larger society. This account seems to be corroborated by the dual role of NGOs in the discourses related to food and fiber. While undeniably contributing to the democratic quality of public discourse, NGOs often appear “not to provide objective and carefully balanced analyses, but rather to raise attention to problems and to pressure governments to do something about it, or to raise funds for their own projects” [21] (p. 414), (*cf.* also [22,23,24]).

The focus of the ordonomic approach is on those discourse distortions that are induced by the perceived misalignment of the individual self-interest and the public interest, the latter of which is typically identified with the common good. This misalignment can take two interrelated forms. First, appealing to promote the common good can blind out the incentives that shape the prudent course of action of self-interested actors; second, situational incentives may exert a strong pressure on self-interested behavior and evoke the impression that contributing to the common good may be much too costly. The misalignment generates two types of dysfunctional moral reasoning, each of which boils down to a seemingly irreconcilable value tradeoff. One type of dysfunctional moral reasoning (“moralism”) gives precedence to public interests while underestimating the role of incentive-based rationality. The other type of dysfunctional moral reasoning (“cynicism”) pleads for abandoning the common good in order to give full sway to the dominant incentives of a given situation. 

The concepts of moralistic and cynic reasoning provide a good illustration of the value conflict underpinning the interaction of NGOs and corporations in the GMO debate. The argumentation strategies of NGOs are often moralistic in that they downplay the legitimate interests of corporations, most importantly the interest in bringing the available scholarly evidence on GMOs to bear on the discourse process. Moralistic reasoning questions the legitimacy of corporations in view of their sheer profit-making orientation. In contrast, cynic reasoning is often attributed to corporations and constitutes the main theme of the institutionalist critique of “corporate hegemony” [25,26,27]. Interestingly, critical institutionalist scholars have documented the degrading effects of the corporate domination of the food and fiber system decades ago [28,29]. In the context of today’s GMO debate, these studies reinforce popular suspicions of the tendency of corporate elites to inflict harm on society and nature for the sake of corporate profits as well as to downplay the moral concerns of corporate critics. It seems clear that both of these types of reasoning distort discourses by promoting a win-lose semantics that assumes the morally troublesome situation to be fixed rather than changeable. Against this backdrop, it may not be exaggerated to characterize both types of moral reasoning as fallacies that increase the likelihood of conflicts instead of promoting the kind of consensual political decision-making that modern democracies require.

Their philosophical weaknesses notwithstanding, both of these fallacies are stable phenomena that are firmly rooted in human nature. In view of their rational ignorance [30], humans pursue rational complexity reduction strategies that prevent them from incurring the cost of searching for, collecting, and analyzing information required for disentangling the misalignments between rational and virtuous behavior. Moralistic and cynic thinking present two subjectively attractive ways of short-circuiting the intellectual efforts that would be necessary for doing so. Hence it has been called a “myth” that voters act rationally [31], which really means that rationally ignorant voters easily fall prey to diverse “myths” in public discourse. 

In addition, recent advances in moral psychology suggest that moralistic and cynic fallacies are stable and rigid to the extent that they stem from moral feelings. Current research in moral psychology testifies to a human tendency to make judgments based on moral feelings rather than rational arguments [9]. If rational arguments are used at all, they provide ex post rationalizations for the decisions that have already been made under the influence of moral feelings (ibid). 

Applying Haidt’s theory of psychologically induced limitations of individual moral behavior to climate ethics, Kasperbauer [32] (pp. 17–18) pleaded for “coordinating group behavior at a policy level”, at which these limitations are more distributed and hence less effective. Given these limitations, appeals to individuals to change their climate-related behavior do not hold out the prospect of success. Haidt’s [9] work informs the ordonomic approach by calling attention to the ambivalent nature of individual moral intuitions. Building on this insight, the ordonomic approach points out the psychological foundations of the moral debates on GMOs and notes the need for policies that would minimize the discourse-distorting consequences of these foundations. It stands to reason that these policies have to capitalize on those individual moral intuitions that are helpful in streamlining the discourse processes and orienting them toward the pursuit of an informed rational consensus. 

### 2.2. Improving the Quality of Discourses 

The ordonomic approach posits that discrepancies between social structure and semantics can put humans in social dilemmas, *i.e.*, situations where individually rational strategies result in socially suboptimal outcomes [17,33,34,35,36]. Social dilemmas feed on moralistic and cynic mental models that eventually result in collective self-damage in spite of individually prudent behavior. Social dilemmas, however, are ambivalent in that they imply a combination of conflicting and common interests. The ordonomic approach draws on this ambivalence and seeks to focus attention on common interests, without denying the existence of conflicting ones. The focus on common interests allows reframing the conflictual semantic categories and mental models in such a way as to realize the latent win-win potential inherent in social dilemmas, in contrast to the win-lose semantics of moralistic and cynic thinking. Ontologically speaking, the source of the tendency to overlook win-win potentials implicit in social dilemmas is the complexity and the systematic role of competition in modern societies [33,36,37]. Morally and emotionally guided decisions short-circuit this complexity and thus fail to recognize the positive-sum game structures underpinning the modern intricate networks of specialization and the division of labor on the local, national, and global scale. Recent studies in the fields of psychology [38] and behavioral economics [39] suggest that moral decision making may be well adapted, in evolutionary terms, to small groups marked by extensive face-to-face interaction [40]. In the larger context of modern complex societies, however, this decision making ends up in moralistic and cynic mental models that exacerbate and perpetuate the social dilemma situations (ibid). 

**Figure 1:**
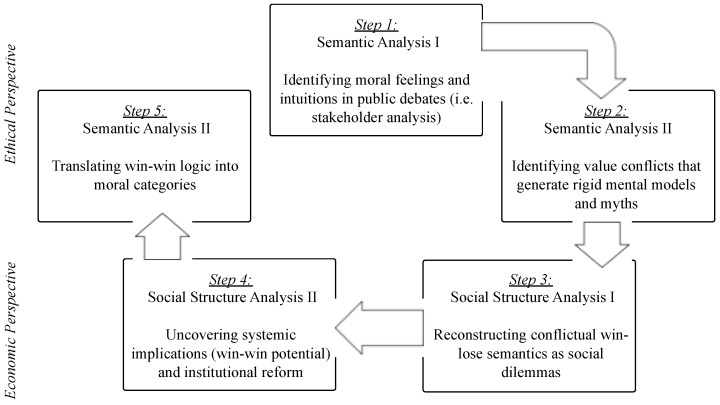
The five-step ordonomic approach to public discourses.

Against this backdrop, the ordonomic strategy of improving the quality of discourses consists of five steps (see Figure 1). The first step involves the identification of emotions and moral feelings, e.g., toward GMOs or small-scale farming [13]. The second step explores whether and how these emotions and moral feelings generate rigid mental models, or “myths”, that block the intellectual activity of discourse participants. In the third step, these mental models are scrutinized for their potential to frame the conflictual win-lose semantics, whether cynic or moralistic, which keeps discourse participants within the bounds of the social dilemma situations. The fourth step seeks to uncover the latent common interests, or win-win potentials implicit within these situations, and to adjust the conflict semantics accordingly. The fifth step is supposed to translate these win-win potentials into (new) moral categories that bring to light the intellectual short-circuiting effects of emotions and moral feelings examined in the first step.

The ordonomic five-step strategy aims at promoting progressive institutional change, *i.e.*, that type of institutional changes that is oriented toward “the continuity of human life and the non-invidious recreation of community through the instrumental use of knowledge” [41] (p. 293). The basic institutionalist concern with progressive institutional change is that it is infeasible in many real-world circumstances as it is blocked by powerful vested interests, which are keen on protecting their privileges [42,43,44]. In the context of GMO debates, the institutionalist concern translates into the potential for the discourse participants to invoke cynic and moralistic reasoning in order to defeat the opposite position, a tendency that includes the detrimental side effect of undermining even well-intentioned endeavors aimed at enlightening the public stance on GMOs. This potential is admittedly considerable. It is probably for this reason that it presently seems impossible to provide uncontestable specific examples of the GMO-related discourses that have already been successfully rationalized along the lines corresponding to the ordonomic approach. Worse yet, current cognitive science is aware of many examples of popular beliefs that persist after having been scientifically refuted [45].

Yet, these critical concerns notwithstanding, there is no reasonable basis for asserting the impossibility of progressive institutional change. The central example here is the rise of science itself. Over the course of human history, science has been continually displacing magic, which has certainly been engrained in conventional emotional intuitions and rigid mental models [41]. Building on Hielscher *et al.*, [42] (p. 779), the rise of science as a progressive institutional change has been possible because it enables, both deliberately and inadvertently, an “inclusive win-win semantics that, by virtue of its very inclusiveness, is capable of transcending the win-lose semantics implicated” in cynic and moralistic reasoning. It is true that there is nothing automatic about this transcending. It may or may not succeed on specific occasions. Still, the normative orientation of the ordonomic approach toward mutual advantage and win-win solutions bears the potential to convince. Even those holding rigid mental models may be tempted to adjust these models if they perceive this adjustment to be in their own self-interest. 

While the ordonomic five-step strategy cannot guarantee success, it can increase its likelihood by accentuating its commitment to ensuring a high quality of public discourse, which, in the last analysis, is a matter of public common interests including most, if not all, discourse participants. Toward this end, the ordonomic strategy is to lay bare and make visible those driving forces that can be commonly acknowledged to be detrimental to rational consensus. The first and the second step of the ordonomic strategy, for example, identify the psychological bases and personal values that influence and stabilize the individual standpoints of discourse participants through processes of habituation and institutionalization. If these processes lower the quality of discourses to the extent that they escape the attention of discourse participants, then making these implicit processes explicit may contribute to undermining their effectiveness. The third step of the ordonomic strategy identifies the win-lose paradigm framing specific discourses, thus creating the basis for discovery of common interests and win-win solutions in the fourth and fifth steps. If the ideas advocated in these steps are valid, they will be appealing to the rational self-interest of most, if not all, discourse participants [46]. 

In practical terms, the ordonomic approach gains credibility by committing itself to strict neutrality with regard to the conflicting standpoints within the discourse. This means that this approach abstains from the moralistic semantics of “rightness” and “wrongness”, as well as of cynic insinuations of vested interests of the concerned discourse participants. Instead, this approach encourages interdisciplinary dialogue, especially between economics and ethics, just as it encourages the interaction between science and mass media. The substantive complexity of the GMO debate makes it difficult to forecast concrete impacts of specific steps of the ordonomic strategy, but it does give hope that even small-scale interventions hold the potential to effect progressive institutional changes on the large scale. 

## 3. Application to Discourses about GMOs: A Conceptual Sketch

This section sketches out a tentative empirically oriented research program that specifies the five-step ordonomic strategy in regard to the phenomenon of moral opposition toward GMOs. This research program combines the tools of ethical and economic analysis in order to expose moralistic and cynic fallacies of dysfunctional mental models (“myths”). 

The first step of the ordonomic strategy can be informed by the classification of subconscious intuitions of the human mind developed by Blancke *et al.*, [47] or Haidt [9].

- Following Blancke and coauthors, the intuitions facilitating the public opposition toward GMOs can be divided in three groups related to (i) essentialism; (ii) teleological and intentional thinking; and (iii) disgust. Essentialist intuitions suggest the inappropriateness of human interference with the allegedly fixed genetic codes; teleological and intentional thinking takes genetic engineering to violate the allegedly beneficial order of nature; and disgust is provoked by the emotional perception of genetically modified food as contaminated [48,49].
- Following the classification proposed by Haidt [9], arguments in favor of GMOs (which are often arguments against the critics of GMOs) usually refer to what Haidt’s “Moral Foundations Theory” identifies as the moral categories of (i) “care/harm” and (ii) “loyalty/betrayal”. Both dimensions belong to a set of at least five “cognitive modules” or “moral taste buds” that emerged in the course of evolution and now trigger fundamental mental programs: “care/harm” provokes compassion for those in need of protection; “loyalty/betrayal” causes “rage against traitors” in the case of offending the established group norms. This is most obvious in popular communication strategies employed by agribusiness firms that aim at discrediting GMO critics as anti-western, anti-capitalist, anti-modernist, or anti-progressive and technophobic.

In terms of a possible empirical methodology, emotions and moral feelings of different actors toward GMOs can be clarified by a discourse analysis of mass media contributions as well as interviews with key actors, such as agricultural associations, agribusinesses and NGOs. This could be supplemented by farmers’ interviews held in representative regions with the purpose of detecting whether and under what conditions farmers would use genetically modified crop varieties. The results of these analyses can be further processed by computer-aided methods such as GABEK^®^ (German acronym for holistic analysis of complexity) in order to reconstruct the meaning of concepts by revealing connections between dominant semantic categories [50].

In the second step, the identified moral intuitions are interpreted as facilitating or even actively contributing to value tradeoffs in public discourses that translate into rigid mental models of agribusiness corporations, consumers, the general public, including NGOs, and policy makers. The basic tradeoff here is between the values of “free enterprise” as advocated by corporations and those of quasi-sacred “natural purity” upheld by the opponents of GMOs. Generally speaking, this tradeoff implies a “win-lose” paradigm of discourse, which will be disadvantageous both for GMO opponents and proponents, because the invoked values are mutually conflicting. This results in moralistic and cynic thinking that mars public mental models, with the undermining effect to inhibit mutual understanding among discourse participants. Operationally, the link between the moral intuitions and value-laden mental models can be detected through expert interviews (as described in step one). The inspiration for these methods may come from e.g., the “market for news model” by Mullainathan and Shleifer [51] or from Swinnen and coauthors that studied “marketing strategies” [21] (p. 415) of NGOs concerned with agriculture-related advocacy.

The third and fourth steps of the ordonomic strategy involve the analysis of social dilemmas to explain discourse distortions. In short, the ordonomic analysis argues that both “moralizing” and “cynic” communication strategies are a prudent reaction of GMO opponents *and* proponents to an uninformed public of rationally ignorant citizens [30]. According to this view, the highly emotionalized discussions on GMOs in Europe are the result of strong incentives faced by discourse participants to feed the public with the easily accessible, but short-circuited positions on genetic improvement technologies. These positions fit well with the moral intuitions of the average citizens. The result is a democratic discourse that produces collectively suboptimal (political) outcomes. One dramatic example, for which both GMO opponents and proponents bear responsibility, is starvation in Africa, which probably could have been prevented or substantially reduced by a more permissive legal attitude to biotechnology [13,52,53]. At the same time, the win-win potentials and the concomitant latent common interests are likewise impressive. According to a comprehensive literature review [47], some varieties of genetically modified corn contain less mycotoxins and thus are healthier than traditional maize; herbicide-resistant crops require less tilling and thus slow down soil degradation; some varieties of genetically modified crops improve insect biodiversity and have strong poverty reduction effects. Here, a key method will be screening and evaluating the literature on the risks and benefits of GMOs.

The fifth ordonomic step calls for identifying institutional and ideational avenues to overcome this situation of collective self-damage. A crucial insight is that the public itself needs to change the dysfunctional incentives faced by discourse participants. Rationalizing the GMO debates, thus, requires institutionalizing fair discourse processes and increasing the GMO debate’s argumentative standards. A further requirement is the translation of rational arguments into moral arguments tied back to the moral categories relevant in public debates. In view of the potential positive effects of GMOs, such moral arguments may refer to the ability of GMOs to enhance human health and dignity, while supporting ecological sustainability. It bears repeating that while the ordonomic approach does not take a position within a given tradeoff, it does identify discourse distortions and ideological biases that hinder democratic rule-setting processes from taking well-informed decisions. In the case of GMOs, an example of discourse distortions is the disregard for the benign consequences of GMOs, as well as the tendencies to discredit discourse participants as anti-progressive or technophobic. A possible ethical methodology for identifying the moral claims underlying public attitudes, corporate strategies and policy initiatives is suggested by the practical syllogism developed by Karl Homann [54]. The practical syllogism is a heuristic framework for deducing normative recommendations for action from the synthesis of normative ideals and empirical implementation conditions. In the GMO debate, it can be used to detect normativistic fallacies that involve translating normative ideals of agriculture directly into recommendations for action while taking no heed of empirical conditions, such as those suggested by scientific arguments. Cynic strategies of discrediting valid ends and concerns can be analyzed in the same fashion.

By working toward overcoming discourse distortions the ordonomic approach seeks to sensitize the public to the whole spectrum of risks and benefits of man-made genetic alterations and thus to demystify popular fallacies, both about GMOs and about their proponents or opponents. Interestingly, this strategy of improving the quality of discourse locates it squarely within the ongoing ethical debate between the agrarian and industrial philosophies of agriculture [55]. While the agrarian philosophy advocates a range of legitimate normative ideals, the industrial philosophy accentuates the modern technological and institutional conditions affecting the way in which these ideals can be implemented. The ordonomic standpoint mediates the debate between these philosophies but is certainly not reducible to the advocacy for GMOs as such.

## 4. Conclusions 

Global discourses about food and agriculture are in a paradoxical state. There is a widespread consensus about the goals of food security as well as economic, social, and ecological sustainability of agricultural production. At the same time, specific strategies for attaining these goals are the object of fierce debates, which are very far from converging towards consensual standpoints. The encompassing rationality related to the sustainability goals of the global food and fiber system seemingly breaks up into multiple partial rationalities, which are unable to establish rational contacts with each other. The present research note has traced this state of affairs to the prevalence of agricultural myths, or rigid mental models, that are impervious both to scientific arguments and to available data. This imperviousness, in turn, is conditioned by the narrow emotional and moral framing of the relevant issues, such as those related to small-scale farming, world hunger, and GMOs. The moral context of the respective discourses opens up new remarkable opportunities for economic ethics, and especially the ordonomic approach introduced here, to make a difference in the lives of billions of people whose wellbeing depends on the global food and fiber system. To this end, the ordonomic approach deconstructs moral arguments in those cases when they are dysfunctional and stand in the way of a search for consensus informed by all relevant arguments. In doing so, the ordonomic approach not only paves the way to identifying the latent win–win potentials within the global food and fiber system but also moves this system closer to the ideal of economic, social, and ecological sustainability. 

## Abbreviations

The following abbreviations are used in this manuscript:

GMO	Genetically modified organism
NGO	Non-governmental organization
GABEK	Ganzheitliche Bewältigung von Komplexität (German acronym for *holistic analysis of complexity*)
